# Abscisic Acid Deficiency Alters Epicuticular Wax Metabolism and Morphology That Leads to Increased Cuticle Permeability During Sweet Orange (*Citrus sinensis*) Fruit Ripening

**DOI:** 10.3389/fpls.2020.594184

**Published:** 2020-12-09

**Authors:** Paco Romero, María Teresa Lafuente

**Affiliations:** Department of Food Biotechnology, Instituto de Agroquímica y Tecnología de Alimentos (IATA-CSIC), Paterna, Spain

**Keywords:** abscisic acid, cuticle, fruit quality, permeability, ripening, transcriptome, transpiration rate, wax morphology

## Abstract

Citrus fruit ripening is coupled with the synthesis and deposition of epicuticular waxes, which reduces water loss during fruit postharvest storage. Although abscisic acid (ABA) is a major regulator of citrus fruit ripening, whether ABA mediates epicuticular wax formation during this process remains poorly understood. We investigated the implication of ABA in cuticle properties and epicuticular wax metabolism, composition, and morphology by comparing the Navelate orange [*Citrus sinensis* (L.) Osbeck] and its ABA biosynthesis-impaired mutant Pinalate in four ripening stages. ABA deficiency had minor effects on cuticle thickness and epicuticular wax load, but correlated with cuticle permeability. ABA content aligned with mostly fatty acids accumulation in both cultivars, and also with specific alkane, terpenoid, and aldehyde constituents in the parental fruit. In turn, cuticle permeability correlated with the fatty acid profile during fruit ripening in the Navelate and Pinalate, and with primary alcohols, terpenoids, and aldehydes, but only in the mutant fruit. Low ABA levels increased the susceptibility of waxes to crack and were lost from the epicuticular layer. The RNA-seq analysis highlighted the differential regulation of a list of 87 cuticle-related genes between genotypes and ripening stages. Changes in the gene expression of the selected genes in both cultivars were consistent with the content of the aliphatics and terpenoid fractions during ripening. The results suggest a role for ABA in the regulation of fatty acid content and primary alcohol composition, and point out the importance of alkane and triterpenoid for controlling water permeance through fruit cuticles.

## Introduction

Citrus fruits are one of the most important crops in terms of production, exportation, and fresh consumption worldwide. Fruit from the genus *Citrus* is grown in more than 140 countries, with an estimated production of over 152 million Tm per year, of which about 85% are consumed fresh.^1^ Therefore, maintaining external fruit quality is crucial in determining the commercial value and consumer acceptance of this crop with its average 16 million Tm export/import trade worldwide (See footnote 1). Dehydration and pathogen attack are the major factors behind the detrimental effects, and the fruit cuticle, and more specifically, the epicuticular wax layer, is a key player in fruit susceptibility to both stresses.

The cuticle is a lipophilic layer synthesized by epidermal cells that covers the surface of aerial plant parts and constitutes the interface between the plant and the environment. Although its major functions include protecting the plant against water loss and from pathogen invasion, it also serves to regulate gas diffusion and dust accumulation, to control temperature fluctuations, to provide mechanical support, and as a signal transduction (Schreiber, 2010; Yeats and Rose, 2013; Aragón et al., 2017). It is composed of a cutin matrix covered and infiltrated with a mixture of cuticular waxes (Kunst and Samuels, 2009; Fich et al., 2016). Waxes are mixtures of aliphatics (e.g., alkanes, alkenes, fatty acids, alcohols, cetones, and aldehydes) that derive from very long-chain fatty acids (VLCFA) and varying proportions of cyclic compounds (e.g., triterpenoids and flavonoids) (Kunst and Samuels, 2009; Bernard and Joubès, 2013). Waxes can be divided into intracuticular waxes, which are embedded in the cutin scaffold, and epicuticular waxes, which form crystals or a smooth film deposited on the outer surface. Research into how these fractions contribute to different cuticle functions has been controversial, mainly when cuticle capacity for water maintenance has been discussed (Kerstiens, 2006; Isaacson et al., 2009; Domínguez et al., 2011a,b). Nevertheless, when focusing on fruit water loss, cutin seems to play a less important role than waxes (Valeska Zeisler-Diehl et al., 2017). Despite intracuticular waxes being commonly considered responsible for reducing water permeability, several studies have correlated epicuticular wax composition with fruit weight loss (Jetter and Riederer, 2016; Trivedi et al., 2019). Indeed, the role of the cuticle in fruit quality and physiology has been largely underestimated, but recent reports have revealed its involvement in postharvest fruit quality (Lara et al., 2014, 2019; Martin and Rose, 2014; Lara, 2018; Tafolla-Arellano et al., 2018).

In citrus, cuticle composition widely varies among species or within cultivars of the same species. It is regulated through fruit and leaf development (Freeman et al., 1979; Sala et al., 1992). Preharvest factors, such as the fruit position on the tree (Sala, 2000) and the application of growth regulators, can also modify its composition and/or morphology (El-Otmani and Coggins, 1985a). Moreover, epicuticular wax composition and structure have an effect on both water loss (Albrigo, 1972; Wang J. et al., 2016) and transport of gases through the cuticle (Ben-Yehoshua et al., 1985; El-Otmani et al., 1986).

Although plenty of information exists about the effect of plant regulators on cuticle formation in Arabidopsis and fruit crops (Ju and Bramlage, 2001; Jeffree, 2006; Knoche et al., 2011; Wang et al., 2011; Martin et al., 2017), knowledge about the hormonal regulation of cuticle biology during citrus fruit ripening remains obscure. The effect of gibberellic acid, 2,4-dichlorophenoxy acetic acid, and ethylene on epicuticular wax composition and/or morphology has been studied as they relate to peel disorders and fruit aging (El-Otmani and Coggins, 1985a,b; Cajuste et al., 2010). However, no information is available about the effect of abscisic acid (ABA) on epicuticular wax content, composition, or morphology in spite of its content increasing during citrus fruit maturation (Lafuente et al., 1997; Romero et al., 2012a, 2019) and dehydration response (Romero et al., 2012b, 2013). Transcriptomic research has suggested that ABA participates in wax biosynthesis based on the correlation of expression data with hormone accumulation during citrus fruit ripening (Wang J. et al., 2016). However, the fact that specific mutants and/or hormone treatments are lacking has left this question open for further investigation.

It has been demonstrated that ABA regulates citrus fruit peel ripening (Romero et al., 2019), which falls in line with the role of this hormone in other nonclimacteric fruit (Leng et al., 2014; Fuentes et al., 2019). This has been possible because of the availability of a fruit-specific ABA-deficient mutant named Pinalate from the Navelate orange [*Citrus sinensis* (L.) Osbeck]. This mutant is not commercialized, but is a good experimental system to better understand the role of ABA in the physiological processes in citrus fruit. Unlike late-ripening and/or stay-green mutants, which present delayed fruit maturation, the Pinalate shows the same maturity index (TSS/acidity) as its parental fruit during fruit maturation despite its yellow-colored fruit (Rodrigo et al., 2003, 2019; Romero et al., 2019). Nevertheless, ABA deficiency in the peel of this fruit has been related to its higher fruit transpirational rate after detachment (Romero et al., 2012b) and also to its attenuated ability to induce stress responses during fruit ripening compared to the Navelate fruit (Romero et al., 2019). “Biosynthesis of cuticular wax” is one of these responses, which suggests the participation of ABA in fruit cuticle structure and composition and, thus, in the differential transpiration rate of these cultivars. However, an exhaustive comparative study of the genes related to wax metabolism, or studying a correlation between them and cuticle properties and composition, has not yet been performed.

Here we test the hypothesis that ABA-reduced levels may cause an alteration to the fruit ripening program that would affect the load, composition, and/or structure of epicuticular waxes with consequences for cuticle metabolism and properties and, hence, for susceptibility to detached fruit dehydration.

## Materials and Methods

### Fruit Materials

Fruit from the Navelate [*Citrus sinensis* (L.) Osbeck] sweet orange and its Pinalate mutant displaying low ABA levels were randomly harvested from adult trees grown in experimental orchards under normal cultural practices and exposed to the same environmental conditions at the IVIA Citrus Germplasm Bank (Moncada, Valencia, Spain). Fruits were harvested during the season until the mature green [MG, harvested 190 days after bloom (DAB)], breaker (Bk, 230 DAB), colored (C, 260 DAB), and full-colored (FC, 330 DAB) ripening stages (Supplementary Figure S1A). We confirmed previous results obtained during different citrus seasons (Rodrigo et al., 2003, 2019; Romero et al., 2019), which indicated that, beside peel color evolution (Supplementary Figure S1A), Pinalate fruit present no delay in internal maturation (TSS/acidity) during ripening. The fruits from both cultivars were harvested on the same day upon each ripening stage. The fruits harvested under each condition (sampling time and genotype) were divided into two groups. In the first group, 15 fruits (three replicates of five fruits each) per condition were included to collect flavedo samples from the total fruit surface, which were frozen and homogenized in liquid nitrogen, and kept at −80°C until analyzed. The second group of each condition consisted of 50 fruits and were used for biochemical and physiological studies. In this second group, three biological replicates for each condition, consisting of 10 fruits each, were stored in the dark and in incubation chambers at 20°C and 60–65% relative humidity (RH) for up to 7 days to induce reproducible water stress and to determine the fruit transpirational water loss rate. The remaining 20 fruits in this second group were divided into four biological replicates of five fruits each and used for the biochemical determinations per condition.

### Transpirational Weight Loss Rate

Fruit surface area was estimated by measuring three fruit diameters per fruit in three replicates of 10 fruits per condition. The stored fruits were weighed daily to determine the amount of water loss. The fruit transpirational water loss rate was calculated as the amount of water loss per surface area and per day. Three biological replicates of 10 fruits each were used per condition.

### Abscisic Acid Analysis

ABA was extracted from 1 g of fresh weight frozen flavedo with 80% acetone containing 0.5 g L^−1^ of citric acid and 100 mg L^−1^ of butylated hydroxytoluene, as previously described by Lafuente et al. (1997). After centrifugation, the supernatant was diluted in three serial dilutions in ice-cold TBS (6.05 g Tris, 8.8 g L^−1^ NaCl and 0.2 mg L^−1^ Mg Cl_2_ at pH 7.8), and three samples for each dilution were analyzed by indirect ELISA. The results are the means of three replicate samples ± SE.

### Cuticle Permeability

The Navelate and Pinalate fruit cuticles were enzymatically isolated as in Isaacson et al. (2009). The permeability of the isolated cuticles was measured by using customized 3D-printed gravimetric chambers (Romero and Rose, 2019). Chambers were folded to expose a known cuticle surface area that acts as the only separation barrier between the water inside the chamber and the environment. Chambers were placed in desiccation containers at room temperature (25°C) and 0% RH. Weight loss was measured daily for up to 7 days, and cuticle permeability was estimated as the weight loss per surface area and per hour. To verify cuticular integrity, cuticles were stained with 0.01% (w/v) aqueous solution of Toluidine Blue O (Sigma) at the end of the assay. Three cuticles from the fruit equatorial zone and 10 fruits per condition were isolated, and 10 replicates per condition were analyzed.

### Light and Scanning Electron Microscopy

Cuticle thickness was determined by light microscopy. Tissue fixation and embedding were performed as in Buda et al. (2009). A solution of Oil Red O (Alfa Aesar) in isopropyl alcohol was applied to the generated 10-μm sections (Martin et al., 2017). The stained slides were visualized under an Eclipse 90i Nikon microscope (Nikon Corporation, Japan) with a 40× objective and the Nis Elements BR 3.2 software (Nikon Corporation, Japan). Cuticle thickness was measured as the distance between the outer cuticle part and the top of the most external epidermal cell by the Fiji Software (ImageJ 1.49q Software, National Institutes of Health, United States). Six pericarp sections from different fruit per condition were analyzed, and 8–10 measurements were taken in each section (50–60 values per condition). To examine the epicuticular wax morphology, peel cubes of 0.2-cm sides from the equatorial zone of five fruits per condition were sliced with a razor blade. Cubes were mounted on aluminum holders, frozen in liquid N_2_, and freeze dried. The freeze-dried materials were sputter-coated with gold film in a sputter coater (Balzers Union Sputtering Device, 25 mA, 300 s) and examined by an SEM (S-4800, HITACHI) instrument at the Microscopy Facility of the SCSIE-UV (Valencia, Spain).

### Cuticular Wax Analysis

The epicuticular wax obtained from intact fruit of known areas was extracted by dipping the fruit for 1 min in two successive chloroform baths, the first containing 100 μg of tetracosane as an internal standard. Wax was derivatized using BSTFA as previously described (Romero and Rose, 2019). After the evaporation of excess BSTFA under nitrogen, wax extracts were resuspended in 100 μl of chloroform and injected into a gas chromatograph (GC) 7890B system (Agilent) equipped with an HP-5MS UI (30 m × 250 μm × 0.25 μm) column (Agilent) and a 5977A simple quadrupole detector (Agilent) at the Gas Chromatography Facility of the SCSIE-UV (Valencia, Spain). Oven temperature was held at 70°C for 2 min before being increased by 10°C min^−1^ up to 200°C, and by 3°C min^−1^ up to 300°C, and then held for 20 min. The injector temperature was 250°C. The identification of most wax components was performed by comparing their relative retention times with those of commercial standards. Computer matching against commercial (Nist, wiley7n) libraries, built by genuine compounds, as well as MS literature data, was also used for identification purposes. Four biological replicates of five fruits each were utilized per condition.

### RNA Extraction and Gene Expression Analyses

Total RNA was extracted from the frozen flavedo samples (Romero et al., 2020a) and treated with Ribonuclease-free DNase (Ambion) following the manufacturer’s instructions. A qPCR analysis was ran to validate the RNA-Seq results and to examine the expression pattern of the selected genes during fruit ripening as in Romero et al. (2020b). cDNA was synthesized by SuperScript III RT (Invitrogen) and Ribonuclease Inhibitor (Invitrogene) following the manufacturer’s instructions. Gene-specific primer pairs, 25 ng of cDNA and SYBR Green 1 Master (Roche Diagnostics), were used to generate relative gene expression data in a LightCycler480 System (Roche) instrument. Genes *ACT* and *TUB* were used to normalize the expression levels of the target genes in the Relative Expression Software Tool.^2^ The employed primers are listed in Supplementary Table S1. Values are the means of three biological replicates with two technical replicates ± SD.

### RNA-Seq, Data Processing, and Normalization

Total RNA integrity was assessed in a 2,100 Bioanalyzer (Agilent) by the RNA 6000 Nano Kit (Agilent). Sequencing libraries were constructed by using three biological replicates of 2 μg of RNA per Navelate and Pinalate flavedo sample for all the ripening stages. Sequencing libraries were generated with the TruSeq Stranded mRNA Library Prep Kit® with PolyA selection for ribo depletion (Illumina) following the manufacturer’s recommendations. Libraries were sequenced on an Illumina NextSeq 500 platform, and 75-bp single-end reads were generated by the Genome Facility of the SCSIE-UV (Valencia, Spain). Raw sequence reads were first checked for quality by FastP (Chen et al., 2018) and FastQC v0.11.8. Clean data were obtained by removing the reads containing only adaptors and filtering sequence reads by at least a mean Q28. Clean sequences were mapped to the reference sweet orange genome sequence (Phytozome release Csinensis_154_v1.1) by using the default settings in the TopHat2 v2.1.0 (Kim et al., 2013) software with the strand-specific alignment type. Seqmonk v1.41 was utilized for quality control, visualization, and quantification. Raw read counts were generated by counting only mapped reads over protein-coding genes with the RNA-seq quantification pipeline by assuming opposing strand specificity. The differential expression analysis of two samples (every three biological replicates) was performed by the edgeR R/Bioconductor package (v3.20.9; Gentleman et al., 2004; Robinson et al., 2010) in the R (v3.4.4) environment (R Core Team, 2020) based on the negative binomial distribution. The resulting values of *p* were adjusted by the Benjamini and Hochberg approach (Benjamini and Hochberg, 1995). The significance of the comparison was determined with an adjusted value of *p* ≤ 0.05, and these genes were considered differentially expressed genes (DEG). The Log_2_ RPM method was followed to estimate the unique gene expression levels. Venn diagrams distribute the number of the DEG that satisfy a cutoff of log_2_ fold change >2. For the multivariate analyses, the Seqmonk tool was used to empirically select highly variable genes by a standard deviation cutoff above 0.7, which resulted in 1,272 genes out of the 25,379 DEG identified among all the comparisons. A principal component analysis (PCA) was conducted with the Log_2_ RPM values and was 3D-plotted by plot.ly. The selected genes were hierarchically clustered by the average linkage method with the Pearson correlation distance metric and were represented by a hierarchical cluster analysis (HCA) and HeatMap created with the MultiExperiment Viewer (MeV 4.9.0) (Howe et al., 2011). The DEG responsible for the PCA and HCA separation were used for gene ontology (GO) categorization purposes. The biological process enrichment analysis of DEGs was implemented by the TopGO (v2.30.0) package (Alexa et al., 2006) with the default “weight01” algorithm. A GO term was considered significantly enriched if more than five DEGs were annotated for that term with a classic Fisher value of *p* < 0.001.

### Statistical Analyses

The statistical analyses were performed using the STATGRAPHICS software.^3^ The data of the parametric variables were subjected to an analysis of variance (ANOVA), and the significance of the differences was determined by Tukey’s test (*p* < 0.05) on the mean values for all the maturity stages and both genotypes. A *t*-test (*p* < 0.05) was used when comparing mutant and parental fruit in a specific maturity stage. For the correlation analyses, the data from the biochemical and physiological parameters were studied by pairs, and linear regression coefficients (R^2^) were calculated by considering both cultivars together or independently.

## Results

### Variations in Fruit Cuticle Properties During Fruit Maturation

**Figure 1:**
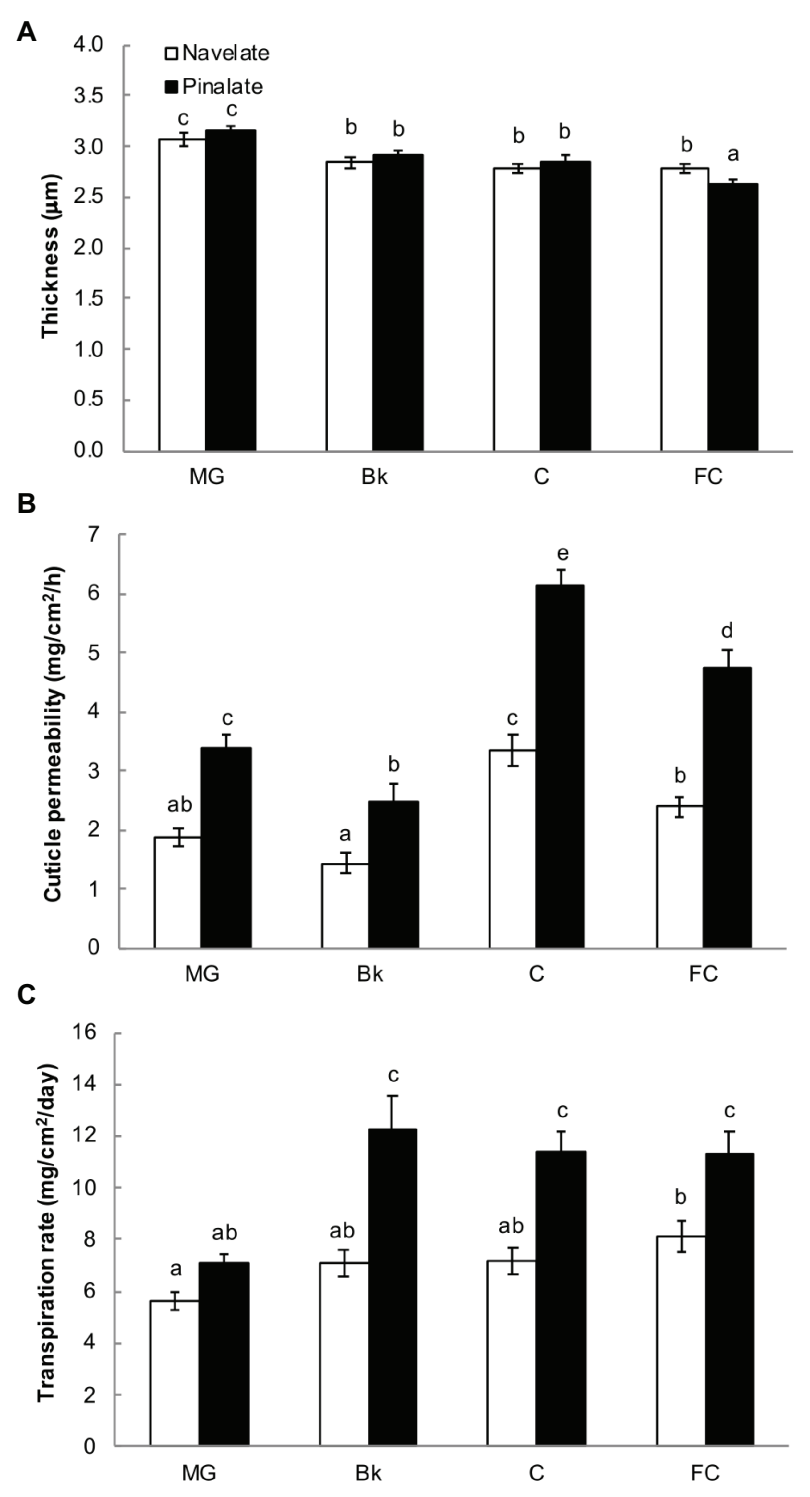
Cuticle properties and fruit weight loss. For cuticle thickness **(A)**, peel sections were stained with Oil Red O, and bars represent the means ± SD of 50–60 measurements for each condition. Cuticle permeability **(B)** was calculated as weight loss per surface area per hour using gravimetric chambers stored for 7 days at 20°C and with 0% RH. Bars are the means ± SD of 10 replicates. **(C)** Transpiration rates of the Navelate and Pinalate fruit, calculated as weight loss per surface area per day from the mature green (MG), breaker (Bk), colored (C), and full-colored (FC) fruit stored for 7 days at 20°C and with 60–65% RH. Bars are the means ± SD of three replicates of 10 fruits each. Different letters indicate the statistical (*p* < 0.05) differences between developmental stages and genotypes according to a multifactor ANOVA analysis followed by a Tukey test (*p* < 0.05) for each studied parameter.

Cuticle thickness continuously decreased in both the Navelate and Pinalate genotypes during the fruit maturation process (190–330 DAB). Significant differences between cultivars were found only for the FC stage, when Navelate cuticles were about 10% thicker than the Pinalate ones (Figure 1A). However, cuticle permeability was higher in the mutant from stage MG to FC. These differences were bigger in the C stage (Figure 1B), when ABA levels peaked in both cultivars, and differences in hormone content were at maximum (Supplementary Figure S1B). Accordingly, cuticle permeability positively correlated with ABA levels when analyzed separately in the Navelate (R^2^ = 0.709) and Pinalate (R^2^ = 0.782) genotypes (Supplementary Table S4). In line with this, the Pinalate fruit obtained a higher fruit transpirational rate than the parental fruit from stage Bk to FC. Differences between cultivars were due to the fact that fruit transpirational rate barely changed in Navelate with fruit ripening, while it sharply increased in the mutant at Bk stage and remained at high levels until FC in this cultivar (Figure 1C). Fruit weight loss showed a low correlation as referred to the ABA content in any cultivar, while it clearly correlated (R^2^ = 0.702) with cuticle permeability (Supplementary Table S4).

### Epicuticular Wax Load and Composition During Fruit Ripening

**Figure 2:**
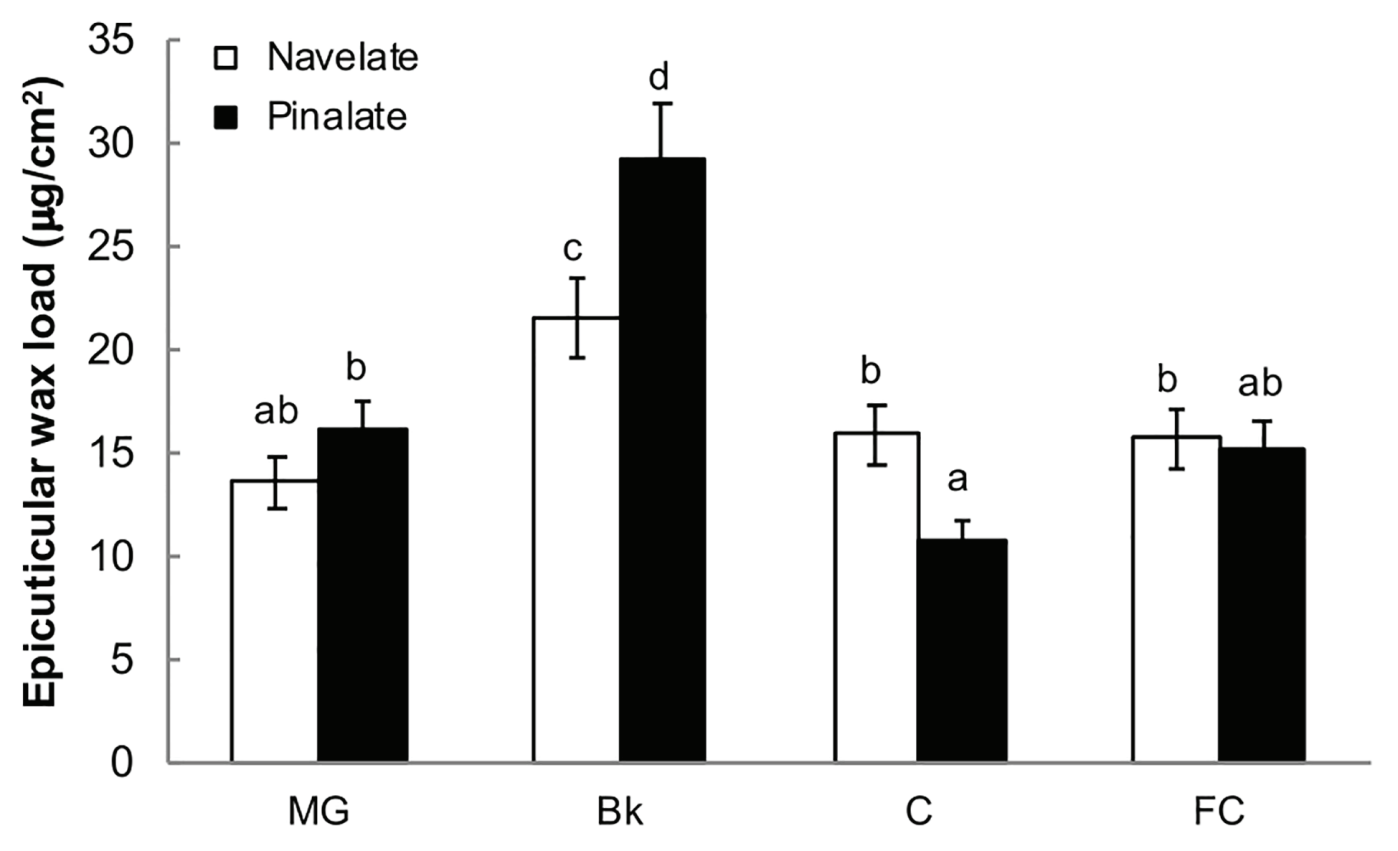
Evolution of epicuticular wax deposition during ripening. The total epicuticular wax load in the Navelate and Pinalate mature green (MG), breaker (Bk), colored (C), and full-colored (FC) fruit. Bars are the means ± SD of four replicates per condition. Different letters indicate the statistical (*p* < 0.05) differences between developmental stages and genotypes according to a multifactor ANOVA analysis followed by a Tukey test (*p* < 0.05).

The total epicuticular wax load per surface area peaked in stage Bk and decreased thereafter to reach stages C and FC at similar levels to those of the MG Navelate and Pinalate fruit (Figure 2). Differences between cultivars were significant in stages Bk and C. Pinalate cuticles had about 40% more epicuticular wax load than Navelate in Bk, while this pattern inversed in stage C (Figure 2). Epicuticular wax load did not correlate with ABA accumulation in any cultivar, while the correlation coefficient between increased cuticle permeability and reduction in total epicuticular wax content was higher in the ABA-deficient mutant than in the parental fruit (Supplementary Table S4).

**Figure 3:**
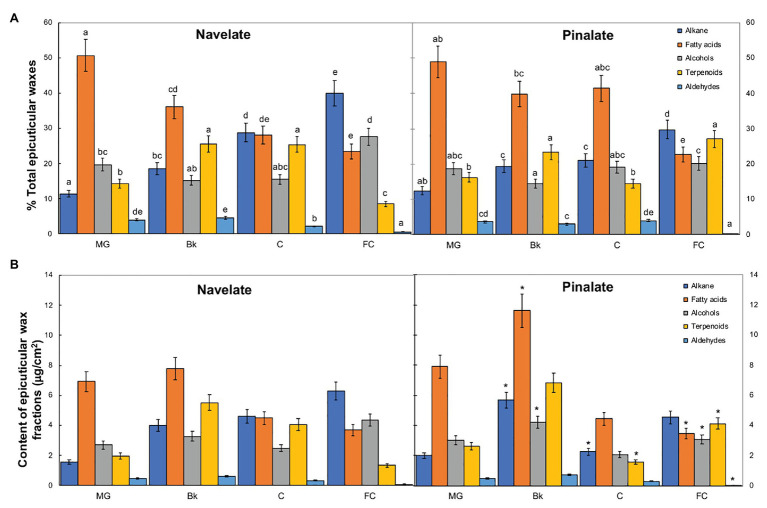
Evolution of epicuticular wax composition during ripening. Percentage (A) and content (B) of the epicuticular wax fractions in the Navelate and Pinalate mature green (MG), breaker (Bk), colored (C), and full-colored (FC) fruits. Bars are the means ± SD of four replicates per condition. **(A)** Different letters indicate the statistical (*p* < 0.05) differences between developmental stages and genotypes according to a multifactor ANOVA analysis followed by a Tukey test (*p* < 0.05) for each wax fraction. **(B)** The asterisks on the Pinalate bars indicate the statistical (*p* < 0.05) differences between cultivars according to a *t* test for each developmental stage.

Studying epicuticular wax composition revealed significant differences between genotypes in stages Bk, C, and FC. In Navelate, the percentage of alkanes increased, while that of fatty acids lowered with ripening. Primary alcohols remained almost steady and peaked only in stage FC, and the percentage of terpenoids increased from MG to Bk and C, and decreased thereafter in FC. The percentage of aldehydes remained very low during fruit ripening and bottomed down in FC (Figure 3). These trends were not completely conserved in Pinalate. So although alkanes also increased continuously with ripening, the percentage of fatty acids remained high from MG to C and lowered only in stage FC. Primary alcohols remained steady throughout the ripening process. Unlike parental cuticles, terpenoid transitory peaked in Bk and later increased in FC. Aldehydes followed a similar pattern to that of the Navelate fruit (Figure 3A). Consequently, the percentage of fatty acids and aldehydes was higher, while that of alkanes and terpenoids was lower in Pinalate than in Navelate in C. In FC, however, the percentages of both alkanes and primary alcohols were lower, but that of terpenoids was higher in the mutant than in the parental fruit. The contents of alkanes, fatty acids, and primary alcohols were higher in Pinalate than in Navelate in stage Bk (Figure 3B).

**Figure 4:**
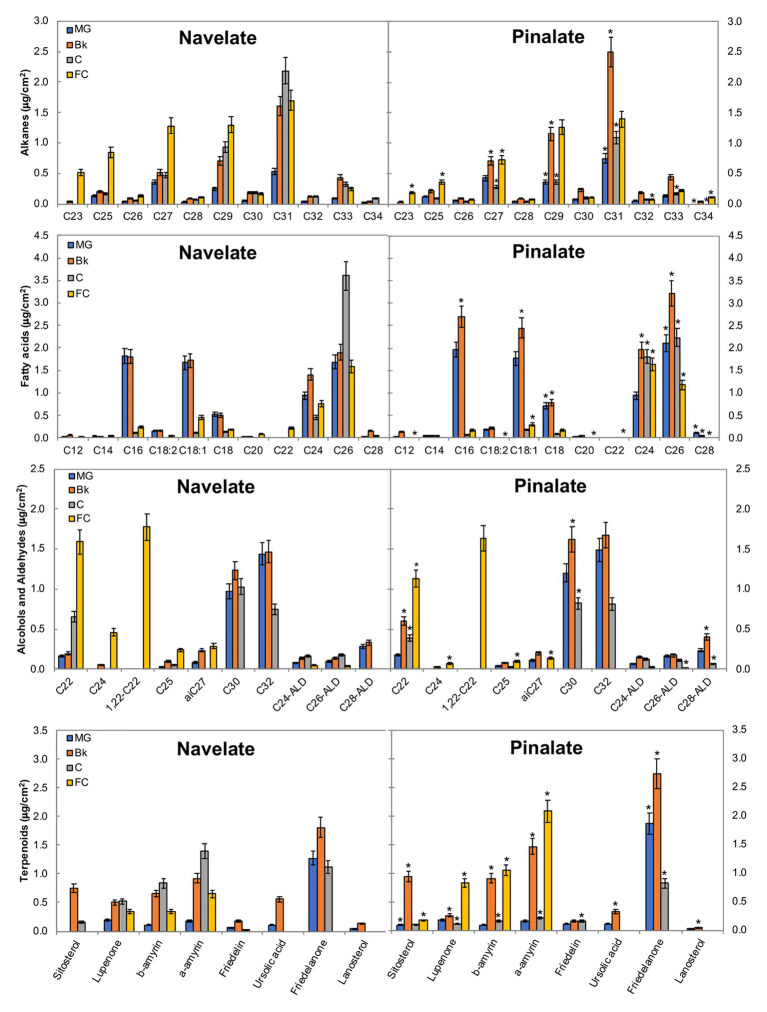
Epicuticular wax constituents during fruit ripening. The total amount of the specific components of the epicuticular wax fractions in the Navelate and Pinalate mature green (MG), breaker (Bk), colored (C), and full-colored (FC) fruit cuticles. Bars are the means ± SD of four replicates per condition. The asterisks on the Pinalate bars indicate the statistical (*p* < 0.05) differences between cultivars according to a *t* test for each developmental stage. Asterisks are also used for those compounds that were detected in only one genotype.

Among long-chain alkanes, heptacosane (C27), nonacosane (C29), and hentriacontane (C31) were the most abundant in both cultivars, while tricosane (C23) and pentacosane (C25) accumulation in Navelate was also noteworthy in FC (Figure 4). In Navelate, these alkanes increased continuously with ripening, whereas this increase was transitory in Pinalate. The most abundant fatty acids in both cultivars were palmitic (C16), oleic (C18:1), stearic (C18:0), lignoceric (C24), and cerotic (C26) acids. In Navelate, all but C26 showed the highest levels in stages MG and Bk, and lowered thereafter. These trends were different in Pinalate (Figure 4). C18:0, however, followed the same accumulation pattern in both cultivars, with higher levels in Pinalate in both MG and Bk. The most abundant primary alcohols were docosanol (C22), 1,22-docosanediol (1,22-C22), triacontanol (C30) and dotriacontanol (C32), and tetracosanol (C24) to a lesser extent in both genotypes. The levels of C22 and C24 alcohols were higher in FC Navelate and that of C30 in the Bk Pinalate fruits. A reduction in aldehyde content in the ABA-deficient mutant was detected, mainly due to the lower hexacosanal levels in FC (C26-ALD) (Figure 4). The most abundant terpenoids were sitosterol, lupenone, α‐ and β-amyrins, and friedelanone. Differences between cultivars in lupenone, α-, and β-amyrin accumulation were evident in stages Bk, C, and FC, while only friedelanone was higher in the Pinalate fruit in MG (Figure 4).

The accumulation of all these specific wax constituents, and that of each epicuticular wax fraction, was analyzed compared to ABA content, cuticle permeability, and fruit weight loss in both the Navelate and Pinalate fruits (Supplementary Table S5). Overall, the regression coefficients markedly improved when both genotypes were studied separately. The regression coefficient between total alkanes and ABA accumulation was higher in the Navelate than in the Pinalate, with the C31 alkane showing the highest correlation (R^2^ = 0.838) in the parental fruit. Very low correlation values were found for the alkane constituents for cuticle permeability in any genotype. However, this fraction highly correlated with fruit weight loss in the Navelate and, to a lesser extent, in the mutant (Supplementary Table S5). Most fatty acid constituents correlated with the ABA content and cuticle permeability in the Navelate and Pinalate fruits, whereas only C24 fatty acid showed a high regression coefficient in relation to the fruit transpiration rate and only in the mutant fruit. For the primary alcohol fraction, a clear correlation was found only for cuticle permeability and only in the mutant fruit (R^2^ = 0.841). It is also worth mentioning that C25 alcohol showed a high regression coefficient (R^2^ = 0.721) with fruit weight loss but only in the Navelate fruit (Supplementary Table S5). Total terpenoids and aldehydes barely correlated with the ABA content, cuticle permeability, or fruit weight loss in any cultivar. Nevertheless, α‐ and β-amyrins showed the highest regression coefficients against ABA in Navelate (R^2^ = 0.728 and R^2^ = 0.546, respectively) and lanosterol in Pinalate (R^2^ = 0.543). In turn, only friedelanone, ursolic acid, and lanosterol correlated to some extent with cuticle permeability, and their coefficients were higher in the mutant fruit (Supplementary Table S5). Of all the aldehydes, C28-ALD correlated with ABA in the parental and with cuticle permeability in both genotypes, but not with fruit weight loss in any cultivar (Supplementary Table S5).

### Differences in Epicuticular Wax Morphology

**Figure 5:**
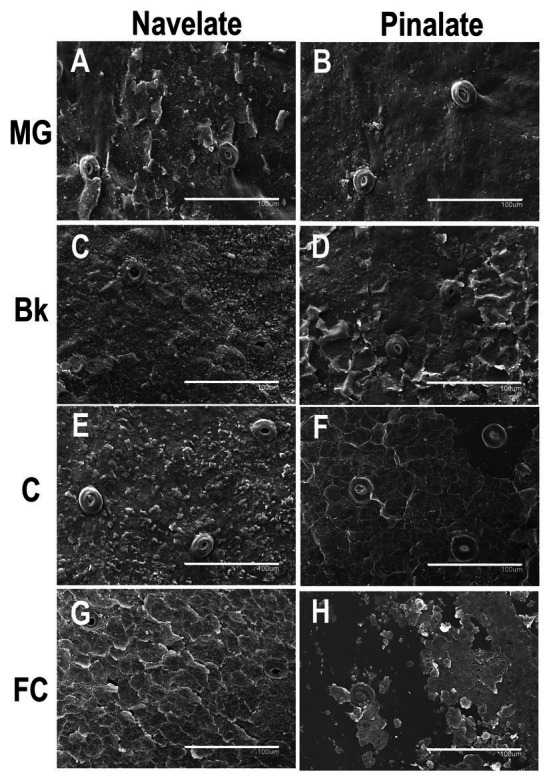
Epicuticular wax morphology during fruit ripening. Scan electron micrographs of Navelate **(A)** and Pinalate **(B)** mature green, Navelate **(C)** and Pinalate **(D)** breaker, Navelate **(E)** and Pinalate **(F)** colored, and Navelate **(G)** and Pinalate **(H)** full-colored fruit ripening stages. The same scale was used in all the micrographs. Scale bars: 100 μm.

The epicuticular wax structure of the MG Navelate and Pinalate orange peel was amorphous, into which platelets and crystalline plates were inserted (Figures 5A,B). Stomata were plugged, and the wax layer around the stomata was not damaged. Structure evolution differed between cultivars during ripening. In stage Bk, homogeneous wax coverage was observed in both cultivars. However, platelets were more abundant in Navelate (Figure 5C). In Pinalate, some plates began to separate from the fruit surface (Figure 5D). Minor differences between the Navelate fruit in stages Bk (Figure 5C) and C (Figure 5E) were noted, although platelet abundance decreased with fruit maturity. In contrast, platelets sharply decreased, and abundant cracks were detected in stage C in the Pinalate fruit (Figure 5F). Epicuticular wax damage increased, and platelets were almost undetectable upon late maturity (FC) in both cultivars (Figures 5G, H), but this effect was more marked in the Pinalate (Figure 5H). In the Navelate, the whole wax layer wax cracked, but no wax deficiency was observed (Figure 5G), whereas separation of the cracked wax was evident in the Pinalate and led to abundant wax coverage loss (Figure 5H).

### Transcriptome-Scale Analysis of Cuticle-Related Genes During Ripening in the Navelate and Its ABA-Reduced Level Mutant Pinalate Fruit

Major changes in gene expression occurred in the Bk Navelate and in FC Pinalate fruit (Supplementary Figure S2A). Differences between the Navelate and Pinalate transcriptional profiles were evident in stages Bk and C (Supplementary Figure S2B), which agrees with the differences in the ABA content (Supplementary Figure S1B). Multivariate analyses (Supplementary Figures S2B,C) validated data repeatability across replications and clustered samples in the ripening stage‐ and genotype-dependent manners. The DEG responsible for this separation (1,272 DEG) were classified into seven GO categories: lipid, cell wall, and carbohydrate metabolism, photosynthetic apparatus, transmembrane transport, hormone and stress response, and general metabolism and developmental processes (Supplementary Table S2). In the present work, “lipid metabolism” regulation was noted, which included biological processes like the biosynthesis of fatty acids and the metabolism of sterols and lipids.

**Figure 6:**
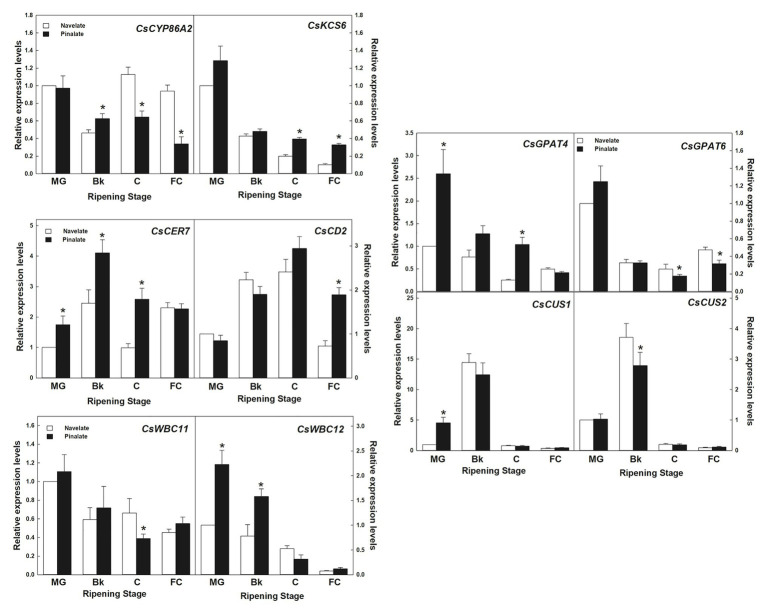
Gene expression analysis of the cuticle-related genes selected from the RNA-Seq transcriptome comparative analysis. Relative transcript abundance for the selected wax and cutin biosynthetic genes, and the cuticle-related transcription factors and transporters belonging to the “lipid metabolism” category differentially regulated in the Navelate (black) and Pinalate (white) flavedo in four ripening stages. For each gene, the transcript levels for all conditions were expressed in relation to the MG Navelate fruit. Data are the mean values of three biological replicates ± SD. The asterisks on the Pinalate bars indicate the statistical differences between genotypes according to a *t*-test (*p* < 0.05) for each ripening stage. These values were also used for validating the transcriptomic data, with an R^2^ value of 0.934 in a linear regression analysis.

In the lipid metabolism category, 10 genes involved in cutin and wax biosynthesis, transport, and cuticle metabolism regulation (Supplementary Table S1) were studied. Expression values further confirmed the reliability of the transcriptomic results by a linear regression analysis (R^2^ = 0.934). Differences between genotypes were detected in specific ripening stages and were not persistent for the whole maturation process (Figure 6). Most decreased with ripening, as observed in cutin biosynthesis genes *GPAT4* and *GPAT6*, *KCS6*/*CER6* elongase, and transporters *WBC11* and *WBC12*/*CER5*. Others, such as *CUS1* and *CUS2*, which are involved in cutin biosynthesis, peaked in Bk and showed very low levels in the other ripening stages in both cultivars. *CER7*, involved in posttranscriptional regulation, and the *CD2* transcription factor peaked in stage Bk and stage C, respectively. The expression levels of *CYP86A2* cutin biosynthesis gene transitory peaked in stage C in the Navelate, but continuously lowered in the Pinalate mutant. It is worth noting that *GPAT4*, *KCS6*, *CYP86A2*, *WBC11*, *WBC12*, *CD2*, and *CER7* (7 of the 10 studied genes) showed significant differences between cultivars in stages Bk and/or C (Figure 6).

**Figure 7:**
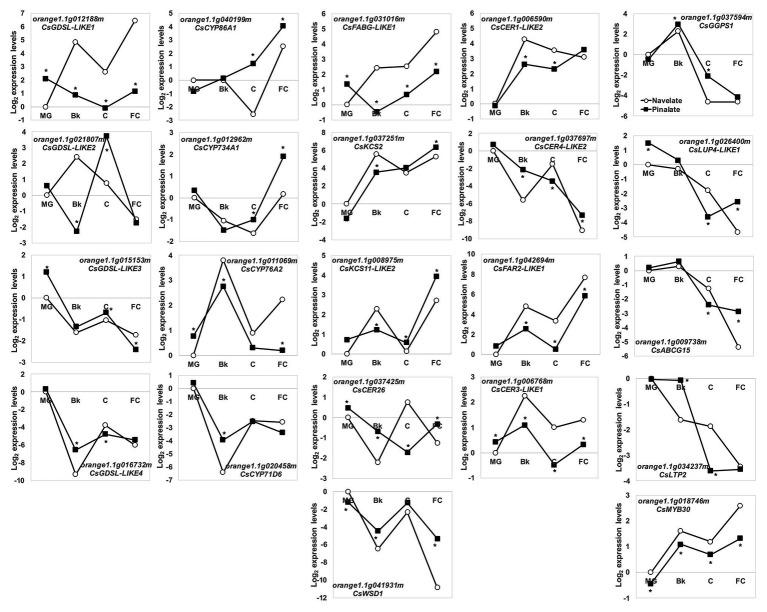
Transcriptional regulation of cuticle metabolism during fruit ripening. Relative gene expression levels of the DEG related to the wax and cutin biosynthesis, and the cuticle-related transcription factors and transporters in the Navelate (white circles) and Pinalate (black squares) mature green (MG), breaker (Bk), colored (C), and full-colored (FC) fruit according to the RNA-Seq data analysis. Values are the means of the log_2_ fold change expression levels of three biological replicates per condition. The expression levels for all conditions were expressed in relation to the MG Navelate fruit. Asterisks indicate the statistical differences according to the edgeR statistical test after modeling the normalized RNA-Seq data to a negative binomial distribution.

This agrees with the most relevant changes in epicuticular wax composition and cuticle properties between the Navelate and Pinalate fruit. For this reason, in a more exhaustive study, we targeted our search for a set of some 100 candidate cuticle-related genes in citrus. Of these, 87 were classified as DEG in our analysis (Supplementary Table S3). To point out the putative participation of ABA in their regulation, we focused on the top five induced/repressed DEGs by comparing both genotypes in stages Bk, C, and FC (30 genes). After removing duplicates, the list was cut to 22 genes, whose expression profiles during fruit ripening are shown in Figure 7. Several members of the GDSL lipase/acyhydrolases and cytochrome P450 families, involved in cutin biosynthesis, were identified. *CsGDSL-LIKE3* and *CsGDSL-LIKE4* bottomed down in Bk in the Navelate and Pinalate fruit, with differences between genotypes in specific ripening stages. *CsGDSL-LIKE1* and *CsGDSL-LIKE2*, however, peaked in the Bk stage in the Navelate fruit. The peak of *CsGDSL-LIKE2* expression in the Pinalate was delayed until stage C, and *CsGDSL-LIKE1* transcript accumulation in the Pinalate remained lower until ripening ended. The expression patterns of the identified CYP450 members were diverse and similar between cultivars, although differences were found in specific maturation stages. *CsCYP76A2* peaked, while *CsCYP71D6* bottomed down in stage Bk in both the Navelate and Pinalate fruit. *CsCYP86A1* and *CsCYP734A1* increased with peel maturation in both cultivars, with the biggest differences between genotypes appearing in stages C and FC. Wax-related genes can be further divided into two groups according to their expression profiles during ripening. The first group included those genes whose expression increased continuously with maturation in both cultivars, which mostly indicated a transitory peak in stage Bk (*CsFABG-LIKE1*, *CsKCS2*, *CsKCS11-LIKE2*, *CsCER1-LIKE2*, *CsCER3-LIKE1*, *CsFAR2-LIKE1*, and *CsMYB30*). The genes included in the second group decreased continuously from MG to FC (*CsLUP4-LIKE1*, *CsABCG15*, and *CsLTP2*), or showed a transitory peak at Bk (*CsGGPS1*) or C (*CsCER26*, *CsWSD1*, and *CsCER4-LIKE2*). The expression profiles in the Pinalate mostly paralleled those of the parental fruit, but numerous differences in transcript accumulation were detected during ripening. In some cases, the Pinalate fruit was unable to develop these transitory peaks in stage Bk (*FABG-LIKE1* and *CsKCS11-LIKE2*) or C (*CsCER26* and *CsCER4-LIKE2*) that were found in the parental fruit.

## Discussion

While cuticle biology has been largely investigated in plants, fewer studies have been performed in fruit despite changes in fruit cuticle metabolism and structure being developmentally regulated (Kunst and Samuels, 2009; Yeats and Rose, 2013; Fich et al., 2016), and its composition and properties determining fruit postharvest behavior by participating in susceptibility to weight loss, pathogen infection, and firmness, among other fruit quality traits (Saladié et al., 2007; Martin and Rose, 2014; Tafolla-Arellano et al., 2018; Lara et al., 2019). Cuticle composition and morphology vary throughout ripening and in response to stresses in citrus fruit (Freeman et al., 1979; El-Otmani and Coggins, 1985a; Sala et al., 1992; Sala, 2000). Transcriptomic approaches have helped to understand the molecular mechanisms underlying its formation (Wang et al., 2014; Liu et al., 2016; Wang J. et al., 2016). However, there is still a gap in knowledge about the role of ABA in the regulation of cuticle properties and composition, even though this hormone is a key regulator of the ripening process and the dehydration response of citrus fruit (Tadeo et al., 2008; Romero et al., 2012a,b, 2019; Zhang et al., 2015; Lado et al., 2018; Terol et al., 2019). We addressed this question using the Navelate orange and its ABA-deficient Pinalate mutant, which presents higher susceptibility to fruit weight loss and pathogen infection during the postharvest (Alférez et al., 2005; Romero et al., 2012b; Lafuente et al., 2019).

### Abscisic Acid Deficiency Increases Fruit Cuticle Permeability by Modifying Epicuticular Wax Composition and Morphology, but Not Total Epicuticular Wax Load or Cuticle Thickness

The Pinalate fruit had more permeable cuticles from early ripening stages than its parental fruit, which was aggravated as maturation progressed, and differences in ABA content increased between cultivars (Figure 1B and Supplementary Figure S1B). The fruit transpirational rate similarly changed, and the Pinalate fruit lost more weight per surface area than the Navelate during ripening after stage MG (Figure 1C). These relations were confirmed by correlation analyses, which revealed high regression coefficients between the ABA content and cuticle permeability for both cultivars separately, and between cuticle permeability and fruit weight loss in the mutant fruit (Supplementary Table S4). In contrast, variations in cuticle thickness were not congruent with differences in cuticle permeability or fruit weight loss, and differences between genotypes were detected only in stage FC (Figure 1A). Therefore, we conclude that cuticle permeability is independent of cuticle thickness, while it is apparently influenced by ABA accumulation. This agrees with studies on cactus pear fruit (López-Castañeda et al., 2010), while an increase in cuticle thickness negatively correlates with fruit weight loss in pepper, plum, and apple fruit (Crisosto et al., 1993; Lownds et al., 1993; Veraverbeke et al., 2001). In tomato, Martin et al. (2017) observed that the ABA-deficient mutant fruits had thicker cuticles and lower transpirational rates than their wild type. From our results, in spite of the correlation between the ABA content and cuticle permeability, an effect of ABA on cuticle thickness along citrus fruit ripening cannot be deduced. This might be related to species but also to the fact that our study focused on fruit ripening covering a range of fruits from 190 to 330 DAB, and cuticle thickness increases at very early developmental stages in citrus fruit, concomitantly with fruit expansion, but then remains constant during the ripening process (Wang J. et al., 2016).

The relation between wax coverage and water permeability has also been largely investigated, but is still controversial. Indeed, cuticle permeability did not correlate with thickness or wax load in a survey of cuticles from 61 plant species (Riederer and Schreiber, 2001), and no apparent relation was found between cuticle permeability and total wax coverage when nine olive cultivars were investigated (Diarte et al., 2019). In contrast, epicuticular waxes have been proposed to be major determinants for water preservation ability in citrus and other fruit crops when constant cuticle thickness is achieved (Knoche et al., 2000; Vogg et al., 2004; Wang J. et al., 2016), which agrees with the fact that stomata are not functional in citrus fruit after detachment (Ben-Yehoshua, 1987). Our results demonstrated that the total wax load did not influence fruit water loss, but correlated with cuticle permeability in the mutant (Figures 1C, 2; Supplementary Table S4). The accumulation patterns of total aliphatics and triterpenoids differed between genotypes during ripening and generated variations in specific ripening stages (Figures 3, 4). Notwithstanding, ABA accumulation did not correlate with any epicuticular wax fraction. However, several specific wax constituents, mainly fatty acids, showed high regression coefficients in both cultivars in relation to ABA content (Supplementary Table S5).

The accumulation of some specific fractions correlated with cuticle permeability and/or the fruit transpirational rate during ripening (Supplementary Table S5). Indeed, we observed bigger differences in cuticle permeability between genotypes in the C and FC fruit stages, which was concomitant with a reduction in n-alkanes in the ABA-deficient mutant (Figure 3). However, cuticle permeability did not correlate with total alkane, but with fatty acids in both cultivars, and also with total alcohols, but only in the Pinalate fruit (Supplementary Table S5). In contrast, fruit weight loss highly correlated with alkane accumulation in the Navelate (Supplementary Table S5). Previous reports have proposed a cuticle architecture model in which low levels of n-alkanes and/or high triterpenoid contents result in a bigger amorphous portion of cuticular wax and, therefore, in higher cuticle permeability and transpirational water loss rates (El-Otmani and Coggins, 1985b; Riederer and Schreiber, 2001; Kosma and Jenks, 2007). Moreover, Grncarevic and Radler (1967) reported that long-chain aldehydes, alkanes, and alcohols are excellent restrictors of the plant transpiration system unlike shorter chain aliphatics and terpenoids, and other cyclic compounds. El-Otmani and Coggins (1985b) linked a lower n-alkane content with higher epicuticular layer permeability in citrus fruit. Interestingly, while the reduction in stage C focused on long-chain alkanes (C27, C29, C31, and C33), in stage FC, it was due to lesser C23, C25, and C27 component accumulation. Moreover, lupenone, and α‐ and β-amyrins were markedly reduced in C, but increased in FC for the Pinalate fruit (Figure 4). These results, together with the regression coefficients between the accumulation of these compounds and fruit weight loss, indicate that n-alkanes and primary alcohols might be more relevant for water retention than triterpenoids. The correlation analyses also indicate that fatty acids play an important role in cuticle permeability in both cultivars, and their accumulation appears to be linked with ABA content during fruit ripening (Supplementary Table S5). Some wax constituents, such as C16, C18, C18:1, and C18:2 fatty acids and the C28 aldehyde, correlate with both ABA and cuticle permeability, but not with fruit weight loss. In other cases, such as short-chain alkanes and most primary alcohols, their accumulation is linked with the fruit transpirational rate, but not with either ABA content or cuticle permeance. Nevertheless, variations in epicuticular wax composition cannot completely explain the differences in the final water permeance through cuticles. This should be explained by the influence of wax morphology as the wax in the Pinalate was more prone to cracking than in the Navelate fruit and mainly in more mature ripening stages (Figure 5). No consistent correlation between chemical composition and wax hardness was found by Freeman et al. (1979), who suggested that the molecular structure of wax constituents could determine wax hardness and influence wax layer loss. Therefore, soft wax is less prone than hard wax to crack or separate (Albrigo, 1972; Sala, 2000; Cajuste et al., 2010). In line with other reports (Wang et al., 2014; Ding et al., 2018), a negative correlation between wax smoothness and the transpiration rate could be deduced from our comparative study (Figure 5). Furthermore, the differences in the wax structure in stages C and FC, when ABA levels most differed between cultivars (Supplementary Figure S1B), suggest that ABA could be a factor that determines epicuticular wax morphology. In fact, El-Otmani and Coggins (1985b) found that when alkane content was high, very little epicuticular wax cracking took place in citrus fruit, which could be the case of the Navelate FC fruit.

The fact that cuticle permeability was higher in the ABA-deficient mutant in stage MG despite the ABA levels between genotypes not differing could be considered counterintuitive because the cuticle transpirational rate correlated with ABA content during fruit ripening (Figure 1; Supplementary Figure S1B; Supplementary Table S4). This result suggests that, first, ABA is not the only factor to regulate cuticle properties upon early citrus fruit ripening, and second, other hormonal control or genetic programs might also participate in cuticle formation in stage MG. Further support for this idea derives from the fact that the percentage of epicuticular wax fractions in stage MG was no different between both genotypes, although we found differences in specific wax components (Figures 2, 3). Hence, the contents of C29 and C31 alkanes, C18 and C26 fatty acids, sitosterol, and friedelanone were higher in the mutant than in the parental fruit in this ripening stage. This indicates that, on the one hand, these compounds might be important components for determining cuticle permeability and, on the other, the participation of ABA in their accumulation seems marginal, specifically in this ripening stage. In fact, C31 alkane and C18 fatty acid were the two wax constituents that most closely correlated with the ABA accumulation profile during ripening in the parental fruit (Supplementary Table S5), which further suggests that ABA-dependent cuticle regulation in stage MG might be minor, probably due to the low hormone levels recorded in this ripening stage (Supplementary Table S1B).

### Abscisic Acid Deficiency Causes Changes in Cuticle-Related Gene Expression

We observed that lipid metabolism was differentially regulated between genotypes in a ripening-dependent manner (Supplementary Figure S2 and Supplementary Table S2). This agrees with, and updates, the previous analyses on the Navelate and Pinalate fruits performed by microarray technology, which covered only two thirds of the *Citrus* genome (Romero et al., 2019). The expression profiles of the cuticle-related genes found in this category, and those acquired from the transcriptomic analysis, indicate two aspects: ABA affects transcript accumulation in a ripening stage-dependent manner; ABA deficiency of the Pinalate fruit causes gene induction/repression for the whole ripening process in most cases compared to parental fruit (Supplementary Table S3; Figures 6, 7).

Cutin biosynthesis in citrus fruit correlates with expansion during fruit development, and thereafter slows down in the fruit ripening phase (Wang J. et al., 2016). Accordingly, *CsGPAT4*, *CsGPAT6*, *CsCUS1*, and *CsCUS2* decreased from stage MG to FC. In contrast, GDSL-like lipase/acylhydrolase and CYP450 members increased or remained at high levels until ripening ended (Supplementary Table S3; Figures 6, 7). A more marked increase in the expressions of *CsGPAT4*, *CsCYP86A1*, and *CsCYP734A1* were observed in the ABA-deficient mutant than in the parental during fruit ripening. Conversely, diminished transcript accumulation of *CsCYP76A2* and *CsGDSL-LIKE1* during ripening, and of *CsCYP86A2* and *CsGPAT6* specifically in FC, occurred in the mutant (Figures 6, 7). Therefore, we cannot rule out the participation of ABA in the different regulations of cutin-related genes in orange fruit during maturation, which would agree with findings in tomato leaves, but not in tomato fruit and Arabidopsis leaves (Kosma et al., 2009; Martin et al., 2017).

Wax biosynthesis initiates in the plastids of epidermal cells with the synthesis of C16 and C18 saturated fatty acids by the fatty acid synthase (FAS) complex. In our analysis, *FABG-LIKE1* was expressed at higher levels in the parental fruit than in the ABA-deficient genotype (Supplementary Table S3 and Figure 7), which might imply that the VLC-acyl-CoA (VLCA) supply for the formation of cutin monomers and wax constituents in the Navelate is higher than in the mutant fruit. In the endoplasmic reticulum, fatty acids are extended to VLCFA by fatty acid elongases (FAE). Of these, β-ketoacyl-CoA synthase (KCS) is a rate-limiting enzyme that determines the carbon chain length of VLCFA, although other elongases like CER26 can also participate in this function (Bernard and Joubès, 2013). The expression profiles of *CsKCS2* and *CsKCS11-LIKE2* were similar between genotypes and were more highly expressed in the ABA-deficient fruit in stages C and FC. In contrast, *CsCER26* expression was higher in the Pinalate during ripening, except for the C stage (Figure 7). These expression patterns differed from those found in other fruits as ABA induced the transcript levels of not only the KCS gene family in apples (Lian et al., 2020) but also of a wax synthase gene in cherry fruit (Correia et al., 2020). Our results agree with the increased accumulation of C16, C18, C18:1, C24, and C26 in the ABA-deficient mutant in stage Bk and with the low C24 levels in stage C in the Pinalate fruit (Figure 4). These findings suggest that ABA might negatively act on the accumulation of these fatty acids. This was further supported by the correlation of several of these compounds with the ABA content and cuticle permeability in both cultivars (Supplementary Table S5).

VLCFA can follow two different pathways: one toward the formation of primary alcohols and wax esters through acyl reduction by CER4 and WSD1, respectively: the other corresponds to the conversion into aldehydes, alkanes, ketones, and secondary alcohols by the decarbonylation pathway, which involves CER1, CER3, and MAH1 (Bernard and Joubès, 2013). In our study, the ABA deficiency caused the induction of *CsWSD1* from stage Bk to FC, which suggests that ABA is a negative regulator of wax ester formation. The expression profiles of *CsCER4*-*LIKE2* and *CsFAR2*-LIKE1, mirrored through ripening, were oppositely affected by the ABA deficiency. Thus, while *CsCER4*-*LIKE2* continuously decreased and was highly expressed in the mutant, *CsFAR2*-*LIKE1* increased during maturation, and transcript levels were lower in the Pinalate (Figure 7). The fact that alcohols C30 and C32 accumulated only until C stage, together with a continuous increase with the ripening of other alcohols up to C27 chain length (Figure 4), suggests that *CsCER4*-*LIKE2* is related to long-chain alcohol synthesis, whereas *CsFAR2*-*LIKE1* might be involved in the lower chain length alcohol formation. This would imply that ABA deficiency alters the composition of the alcohol fraction in such a way that the compounds with long chain lengths gradually reduce, while those with shorter carbon chains are induced as ripening progresses. This falls in line with previous research that has revealed that FAR genes can be regulated by ABA treatment or abiotic stresses in an ABA-dependent manner to, hence, modify the alcohol composition of cuticles in wheat leaves (Wang et al., 2015; Wang M. et al., 2016).

In previous reports, *CER1* has been proposed to encode VLCA reductase by catalyzing the reduction of VLCA to aldehydes and the decarbonylation of aldehydes to alkanes (Aarts et al., 1995; Rowland et al., 2007). *CsCER3* is proposed as a candidate gene for alkane formation from VLCA (Liu et al., 2015). We found that *CsCER1-LIKE2* and *CsCER3-LIKE1* expressions increased with ripening in both genotypes, but remained at lower levels in the Pinalate (Figure 7). This agrees with the ABA-activated induction of CER1 and the consequent increase in alkane production in wheat leaves (Li et al., 2019). Our gene expression results are consistent with the lower content of aldehydes in the Pinalate than in the Navelate in FC fruit (Figure 3B), and also with the reduction in n-alkanes from stage Bk to the end of ripening in the mutant, which supports a role for ABA in the regulation of these fractions (Supplementary Table S5).

CsGGPS1 provides the backbone for triterpenoid formation, and *CsLUP4-LIKE1* encodes a β-amyrin synthase involved in triterpenoid biosynthesis. These genes were downregulated as ripening progressed, which agrees with previous reports about other citrus cultivars (Liu et al., 2016). Differences between cultivars in the accumulation of lupenone, and α‐ and β-amyrins match the expression profile of *CsLUP4-LIKE1* (Figures 4, 7) and correlate to some extent with the ABA profile during ripening (Supplementary Table S5). This scenario suggests that *CsLUP4-LIKE1* is regulated by ABA and is responsible for the accumulation of these triterpenoids in citrus fruit during ripening.

After synthesis, ATP-binding cassette (ABC) transporters and other proteins, such as nsLTP that facilitate lipid transfer between liposomes, are involved in exporting wax components to the apoplastic environment (Luo et al., 2007; Bird, 2008). In our study, *CsWBC11*, *CsWBC12*, *CsABCG15*, and *CsnsLTP2* decreased with ripening in both cultivars, while *CsWBC12* and *CsnsLTP2* were induced in the Pinalate in the Bk stage, and the *CsWBC11*, *CsnsLTP2*, and *CsABCG15* expression levels were lower in the mutant fruit than in the parental fruit in stage C (Figures 6, 7). These differences partially explain the differences in wax load between cultivars in stages Bk and C (Figure 2) and suggest that ABA might regulate the export of wax components to the extracellular matrix.

Among the genes that regulate wax metabolism, our analyses highlighted the *CsMYB30* transcription factor and the *CsCER7* post-transcriptional modulator. MYB30 has been reported in plants to act as a regulator of four genes that encode FAE enzymes in response to pathogen attack (Raffaele et al., 2008). *CsMYB30* expression levels in the Pinalate were lower than in the parental fruit during ripening, as well as those of *CsCER1-LIKE2*, *CsCER3-LIKE1*, and *CsCYP86A2*, which paralleled the *CsMYB30* transcript levels and are proposed as targets for this transcriptional regulator (Figures 6, 7). This indicates that *CsMYB30* is involved in cuticular wax biosynthesis regulation, and the ABA deficiency attenuates its expression. CER7 is a core subunit of the RNA-degrading exosome complex in relation to the posttranscriptional regulation of cuticular wax biosynthesis in Arabidopsis (Hooker et al., 2007) through the induction of *CER3*. We found that *CsCER7* was induced in the Pinalate fruit from stage MG to C, whereas *CsCER3-LIKE1* was continuously repressed in the ABA-deficient mutant compared to the parental fruit (Figures 6, 7). Despite these results envisaging an ABA-dependent regulation of *CsCER3-LIKE1* in citrus, its regulation by CsCER7 is less clear and merits further research.

## Conclusion

These results provide valuable data to link differences in the accumulation of specific metabolites with changes in the transcript levels of cuticle-related genes in two cultivars differing in their endogenous ABA levels and fruit transpirational rates. Our study demonstrates that cuticle permeability correlates with the ABA content during ripening, but not with cuticle thickness or total epicuticular wax load. Furthermore, differences in ABA levels affect the epicuticular wax layer morphology and relate to the accumulation of specific wax constituents. ABA deficiency affects cuticle metabolism in transcriptional terms, but not over the entire biosynthetic pathway, rather in accordance with a gene-specific basis, which explains the large differences observed in the composition of wax fractions and the lack of correlation between hormone content and the total wax load.

## Data Availability Statement

Data supporting the findings of this study are available in this publication and its Supplementary Materials published online. The datasets used for the transcriptomic study can be found at the NCBI repository (BioProject ID PRJNA674975).

## Author Contributions

PR performed the research, analyzed the data, and wrote the original draft with contributions by MTL. MTL and PR conceived the project and accepted the final manuscript.

### Conflict of Interest

The authors declare that the research was conducted in the absence of any commercial or financial relationships that could be construed as a potential conflict of interest.

## Footnotes

^1^http://www.fao.org/faostat

^2^http://rest.gene-quantification.info

^3^www.statgraphics.com
