# Supplementary material for: Abscisic Acid Deficiency Alters Epicuticular Wax Metabolism and Morphology That Leads to Increased Cuticle Permeability During Sweet Orange (Citrus sinensis) Fruit Ripening
Source: Front Plant Sci. 2020 Dec 9;11:594184. doi: 10.3389/fpls.2020.594184 (PMC7755607; doi:10.3389/fpls.2020.594184)
Supplement: Supplementary file 2 [file Data_Sheet_2.pdf]

Table S1. Primers used for the gene expression analyses.

Citrus ID	Short name	Forward Sequence (5' - 3')	Reverse Sequence (5' - 3')	Amplicon size (pb)
orange1.1g024218m	*CsCD2*	GGCGTATTGCCAACATTCTT	CCTCACGCTCAAAAGTCTCC	204
orange1.1g043337m	*CsCER7*	GGTCGCAACCCTTTTGAATA	GGATCAGCCATTGGAGAGAA	188
orange1.1g017552m	*CsCUS1*	AACAATGACTACCTTGCCACCACT	CTGAAAATACTCGATCTGCTTGCT	288
orange1.1g017181m	*CsCUS2*	CAGAAAAATTGGTCAAACCGTCTTC	GCTGAGATTCATAGGGGTCATATAGT	286
orange1.1g009235m	*CsCYP86A2*	CAAACACGTCGCACTCAACT	TCAAACCCTAACGGCTCATC	289
orange1.1g010860m	*CsGPAT4*	GCAAAGTCTCGTGCGTTACA	TGCGAACAAAGCACTGAATC	200
orange1.1g018906m	*CsGPAT6*	TATGCAACCATCGCACTGTT	GGCAGATCACCAAGTCACCT	198
orange1.1g011007m	*CsKCS6*	ACTTCATGTCCAAGCCAAGG	GCAGGTCTCTTCACCGAGTC	185
orange1.1g005142m	*CsWBC11*	GATTTCTACCAAACTTCTCAGCACTCG	ATTGTAACCAGTTCCCACGTTCAGAT	252
orange1.1g046969m	*CsWBC12*	AGCAAAGTTGGCTGTGGAGT	TCTCAGCTGTTGCCATGTTC	191
orange1.1g013062m	*CsACT*	TTAACCCCAAGGCCAACAGA	TCCCTCATAGATTGGTACAGTATGAGA	176
orange1.1g013062m	*CsTUB*	GCATCTTGAACCCGGTAC	ATCAATTCGGCGCCTTCAG	158

Table S2. Gene ontology categorization of the DEG responsible for the separation of genotypes and developmental stages in the HCA and PCA analyses.

Category	GO Term (Biological Process)	GO Code	*p value*
**Cell wall metabolism**			
	tryptophan biosynthetic process	0000162	0.00140
	cellular glucan metabolic process	0006073	0.00929
	asparagine biosynthetic process	0006529	0.03915
	S-adenosylmethionine biosynthetic process	0006556	0.03930
	cell wall macromolecule catabolic process	0016998	0.00075
	cell wall modification	0042545	0.03177
**Lipid metabolism**			
	lipid metabolic process	0006629	0.02120
	fatty acid biosynthetic process	0006633	0.02010
	lipid biosynthetic process	0008610	0.02625
	sterol metabolic process	0016125	0.00029
**Carbohydrate metabolism**			
	polysaccharide catabolic process	0000272	0.01540
	carbohydrate metabolic process	0005975	0.04310
	trehalose biosynthetic process	0005992	0.04640
	GDP-mannose biosynthetic process	0009298	0.02705
	inositol catabolic process	0019310	0.00082
	glucosinolate catabolic process	0019762	0.00179
	cellulose biosynthetic process	0030244	0.01271
**Photosynthetic aparatus**			
	photosynthesis, light harvesting	0009765	0.02610
	photosynthesis	0015979	0.00745
	photosystem II stabilization	0042549	0.04355
**Transmembrane transport**			
	amino acid transmembrane transport	0003333	0.04120
	ion transport	0006811	0.02290
	drug transmembrane transport	0006855	0.03410
	purine nucleobase transport	0006863	0.03540
	metal ion transport	0030001	0.00379
	glycolipid transport	0046836	0.01107
	transmembrane transport	0055085	0.02005
	potassium ion transmembrane transport	0071805	0.01389
	sulfate transmembrane transport	1902358	0.04625
**Hormone and Stress responses**			
	chitin catabolic process	0006032	0.00089
	protein phosphorylation	0006468	0.01985
	protein dephosphorylation	0006470	0.03470
	proline catabolic process	0006562	0.00939

spermine biosynthetic process	0006597	0.00280
defense response	0006952	0.00660
spermidine biosynthetic process	0008295	0.00103
response to water	0009415	0.03177
SOS response	0009432	0.00009
response to wounding	0009611	0.08333
response to salt stress	0009651	0.00078
jasmonic acid metabolic process	0009694	0.00003
response to abscisic acid stimulus	0009737	0.00010
induced systemic resistance	0009864	0.00034
defense response to bacterium	0042742	0.04481
defense response to fungus	0050832	0.01018
**Metabolism and Development**		
cell morphogenesis	0000902	0.02180
DNA replication initiation	0006270	0.01934
protein retention in ER lumen	0006621	0.00610
exocytosis	0006887	0.03560
microtubule-based process	0007017	0.00327
microtubule-based movement	0007018	0.00032
signal transduction	0007165	0.00075
metabolic process	0008152	0.00082
cell proliferation	0008283	0.00179
deoxyribonucleoside diphosphate metabolic process	0009186	0.01874
embryo development	0009790	0.03332
positive gravitropism	0009958	0.00197
coenzyme A metabolic process	0015936	0.00355
protein ubiquitination	0016567	0.00032
developmental process	0032502	0.01874
mitochondrial respiratory chain complex	0034551	0.02306
nitrate assimilation	0042128	0.02705
recognition of pollen	0048544	0.00051
oxidation-reduction process	0055114	0.02005
nitrile biosynthetic process	0080028	0.02504

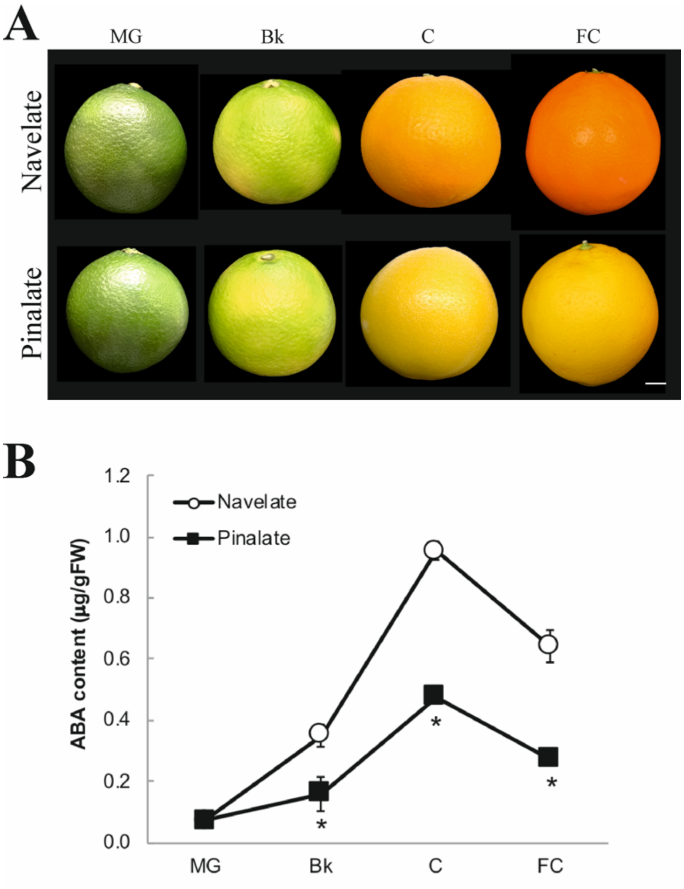

Figure S1. Color evolution and ABA accumulation during fruit ripening. (A) Representative photographs of Navelate and Pinalate fruit along the four ripening stages analyzed in this study: Mature green (MG), breaker (Bk), colored (C) and full colored (FC) fruit. Scale bar: 1 cm. (B) ABA content in the flavedo of Navelate and Pinalate fruit along ripening. Values are means ± SD of 3 replicates for each condition. Asterisks indicate statistical ($P < 0.05$) differences between cultivars according to a *t-test* for each developmental stage.

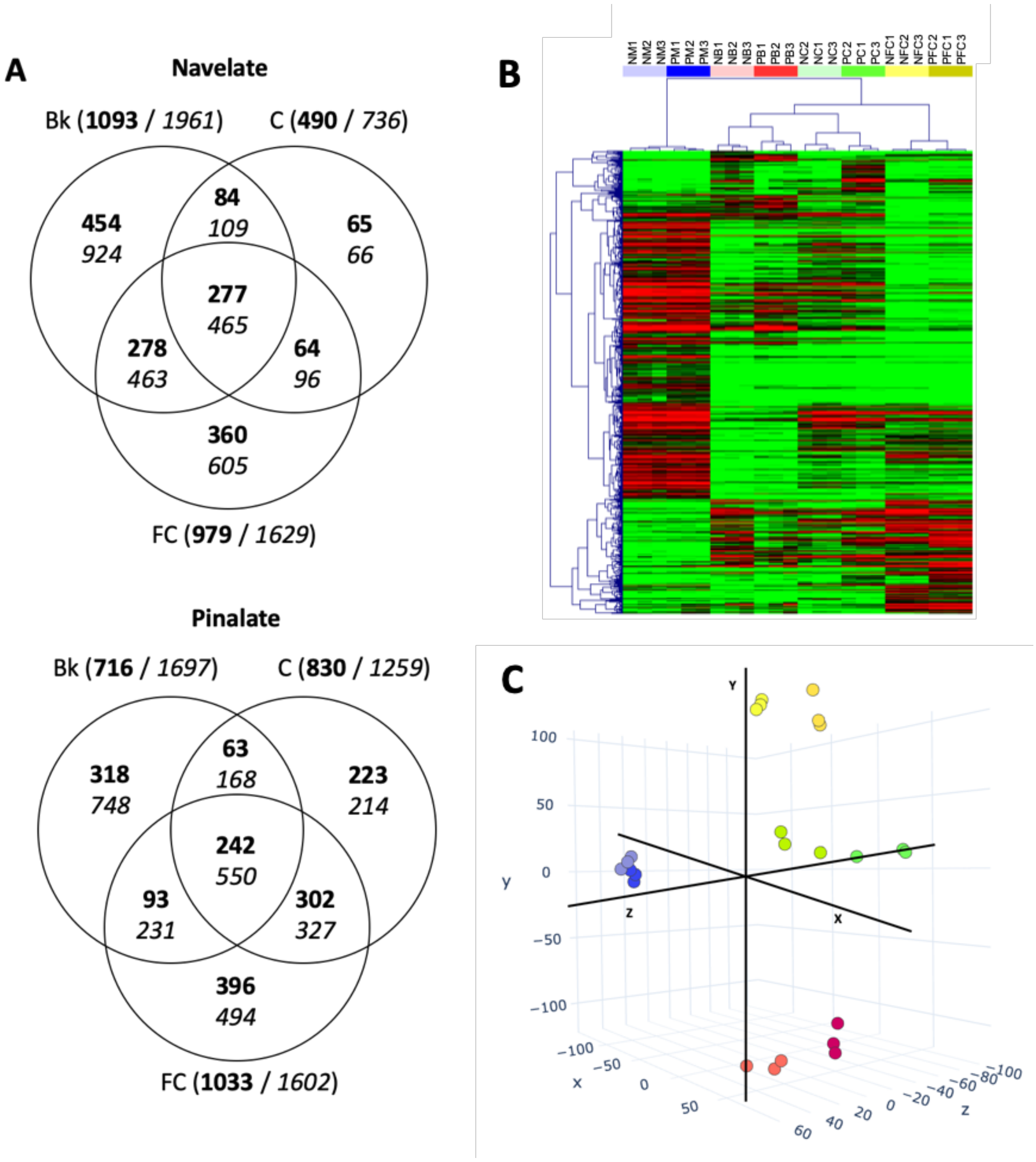

Figure S2. Transcriptome analysis during peel ripening. (A) Venn diagrams of Navelate and Pinalate fruit showing the distribution of differentially expressed genes (DEG, edgeR, BH p-value adjustment α=0.05) satisfying a cutoff of Log2 FoldChange > 2 for the comparisons between breaker (Bk), colored (C) and full colored (FC) Navelate (N) and Pinalate (P) fruit respect to their mature green (MG) developmental stage. Inductions and repression are indicated in bold and italics, respectively, and the total number of induced and repressed DEG for each developmental stage are indicated in brackets. (B) Hierarchical Cluster Analysis (HCA) and Heatmap large-scale transcriptional profiles, and (C) Principal Component Analysis (PCA), based on the 1272 DEG satisfying a cutoff of STDEV > 0.7 for

all conditions represented in Venn diagrams. The colors in HCA for each condition are consistent with those in PCA. The three axes in PCA account for 92.1% of the total variance among genotypes and developmental stages. Heatmap colors vary from light green (repression) to dark red (induction). Three biological replicates from each condition were used for the analyses.
